# RETRACTION: Dermoscopy and Reflectance Confocal Microscopy Finding of Different Anatomic Sites of Langerhans Cell Histiocytosis

**DOI:** 10.1111/srt.70231

**Published:** 2025-09-18

Zhang G, Wang X, Wang Y, et al. Dermoscopy and reflectance confocal microscopy finding of different anatomic sites of Langerhans cell histiocytosis. *Skin Res Technol*. 2024;30:(1):e13584. doi:10.1111/srt.13584

The above article, published online on 18 January 2024 in Wiley Online Library (wileyonlinelibrary.com), has been retracted by agreement between *Skin Research and Technology*; and John Wiley & Sons Ltd. The article was accepted for publication following an insufficient peer review process that does not meet the standards of the journal's peer review policy. Furthermore, the article lacks a clear study design and the conclusions presented are not supported by the presented data. Accordingly, the article has been retracted. The authors have been informed of the retraction decision but were unavailable for final confirmation.

